# Optical Redox Imaging Predicts Post-Loading Cartilage Mitochondrial Membrane Potential

**DOI:** 10.1007/s10439-025-03784-1

**Published:** 2025-06-27

**Authors:** Jingyi Wang, Greta E. Scheidt, Corinne R. Henak

**Affiliations:** 1 Department of Mechanical Engineering, University of Wisconsin-Madison, 3031 Mechanical Engineering Building, 1513 University Ave, Madison, WI 53706 USA; 2 Department of Biomedical Engineering, University of Wisconsin-Madison, Madison, WI USA; 3 Department of Orthopedics and Rehabilitation, University of Wisconsin-Madison, Madison, WI USA

**Keywords:** Cartilage, Mechanobiology, Optical redox imaging, Mitochondrial membrane potential

## Abstract

**Purpose:**

Disrupted cellular redox balance is associated with various diseases, including osteoarthritis. Although mitochondrial (MT) membrane potential is a proxy for redox balance, the translational potential of this method is limited by exogenous dye. Therefore, the objective of this study was to predict changes in MT membrane potential in response to mechanical loading using dye-free optical redox imaging (ORI). A secondary objective was to determine the effect of loading on ORI metrics.

**Methods:**

Full-thickness porcine cartilage strips were subjected to tensile loading at one of two strain rates (1.00 s^−1^ or 0.10 s^−1^). ORI was done before, immediately after, and 30 minutes after loading. MT membrane potential was then measured using fluorescent dye. A generalized linear mixed-effects model (GLMM) tested main effects (ORI metrics, loading vs. control, loading rate, post-loading time, zone) and their interactions in prediction of MT membrane potential. Significant predictors were retained in a new GLMM that was trained using 70% of the dataset and evaluated using the remaining 30%. Two separate GLMMs evaluated the main effects on ORI metrics.

**Results:**

In the GLMM using MT red/green ratio as the dependent variable, ORI metrics, loading rate, and loading vs. control were significant main effect. GLMMs to predict MT red/green from ORI that retained significant main effects resulted in an average difference between predicted and actual values of 7.07%. When analyzing the effect of loading vs. control, loading rate, and zone on ORI metrics, only loading rate showed significance.

**Conclusion:**

ORI can predict MT membrane potential measured by fluorescent dye and has the possibility to be developed as a clinical tool to evaluate cartilage redox balance.

**Supplementary Information:**

The online version contains supplementary material available at 10.1007/s10439-025-03784-1.

## Introduction

Disruption of redox balance, the balance between reductive and oxidative reactions, is both an indicator and a driver of various diseases including osteoarthritis (OA) [1, 2]. OA is a prevalent degenerative joint disease with cartilage degradation as a central aspect. Redox imbalance often results from a relative increase in oxidants in the intracellular environment. One class of oxidants that can over-accumulate is reactive oxygen species (ROS), highly reactive molecules that are normally produced at low levels to regulate cell function [3, 4]. Whether oxidants accumulate by overproduction or by reduced consumption, the end result is oxidative stress and eventually cellular and tissue damage. For example, ROS degrade cartilage by serving as the secondary messenger in matrix metalloproteinase production and by downregulating extracellular matrix production [4]. Given the role of redox imbalance in OA, measuring cartilage redox balance is of interest as a method to understand disease states and evaluate therapeutic strategies.

Mechanical stimuli play a crucial role in the regulation of the cartilage cellular environment, including in regulating redox balance. Mechanical loading can cause negative consequences. For example, abnormal mechanical stress mediated production of nitric oxide (NO) [5], while repeated compression to 1 MPa increased NO production and suppressed respiration [6]. Conversely, cyclic compressive loading in the physiological range (20% strain) increased endogenous antioxidant gene expression [7], demonstrating the positive effect of mechanical stimuli in the physiological range. While compression is the most extensively studied, cartilage redox balance is responsive to other loading modes as well: hydrostatic pressure on chondrocytes increased ROS production [8], and biaxial cyclic tension suppressed NO production induced by IL-1β [9]. In addition to whole-depth responses to mechanical stimuli, prior studies have shown that cartilage mechanoresponses vary between the superficial, middle, and deep zones. In full-thickness cartilage explants subjected to low strain-rate compression, most dead cells were in the middle zone, while most were in the superficial zone when subjected to high strain-rate compression [10]. The superficial zone is generally considered more responsive to stimuli, such as mechanical injury [11].

Mitochondria (MT) function is closely tied to redox balance and is straightforward to measure; therefore, it is often used as a proxy measure. MT are the primary site of oxidant production, including ROS production, and are damaged in response to oxidative stress [12]. Because the MT transmembrane potential gradient is essential for MT homeostasis [13, 14], fluorescent probes that evaluate MT membrane potential are commonly used to measure changes in MT function and thereby disruption in redox balance [15]. A previous study showed that the red/green fluorescence intensity ratio (R/G ratio, corresponding to polarized/depolarized MT, respectively) of OA chondrocytes was lower than that of normal chondrocytes, indicating depolarized membrane potential and impaired MT function in OA chondrocytes [16]. MT dysfunction induced by superoxide treatment in wild-type chondrocytes increased MT depolarization and decreased oxygen consumption rate [17], demonstrating that the effects of ROS can act directly on MT. Similarly, MT dysfunction can be induced by mechanical overload, as observed in bovine cartilage explants after impact injury [18]. MT membrane potential and other measurements, such as fluorescent labeling of specific ROS and fluorescent probes for mitochondrial function, provide measurements of cellular redox balance, but they remain challenging to translate because of their use of exogeneous dye.

Endogenous fluorophores are an attractive alternative to exogeneous dyes. Optical redox imaging (ORI) utilizes the autofluorescence generated by flavin adenine dinucleotide (FAD) and nicotinamide adenine dinucleotide and its phosphate (together termed NAD(P)H) to capture redox balance [19]. Previous studies from our lab have shown that ORI metrics were responsive to mechanical stimuli [20] and correlated with cartilage histopathology score [21]. How ORI metrics relate to other well-established measures of redox balance such as MT depolarization, however, is unknown, leaving a fundamental gap that limits its clinical application in cartilage.

Therefore, the primary objective of this study was to establish the relationship between ORI metrics and MT membrane potential measured using a fluorescent probe. The hypothesis was that tensile loading-induced changes in MT membrane potential could be predicted by ORI metrics. The secondary objective was to determine the effects of tensile loading on cartilage redox balance as measured by ORI. The secondary hypothesis was that the response of ORI metrics to mechanical loading would be rate- and zone-dependent.

## Methods

### Sample Preparation

Porcine right patellae were harvested within 2 hours after euthanasia for other purposes and stored in phosphate-buffered saline (PBS) with 1% penicillin-streptomycin (Quality Biological) during transportation to the laboratory. Full-depth rectangular cartilage explants were collected using a scalpel and manually cut into 0.66 ± 0.17 mm by 10 mm full-depth strips using a razor blade. The thickness of each sample was measured using a caliper. All samples were trimmed to fit in the loading set-up with fixed initial length of 10 mm. Cartilage strips were placed in 12-well plates and cultured in chemically defined chondrocyte medium (1X DMEM with high glucose, 50 μg/mL L-Ascorbic acid 2-phosphate salt, 50 μg/mL L-proline, 1% insulin-transferrin-selenium (ITS), 1 mM sodium pyruvate, 100 nM dexamethasone, and 1% penicillin-streptomycin) in 5% O_2_ and 5% CO_2_ at 37 °C.

In total, 37 pairs of strips were collected from 4 pigs (approximately 4–5 months old, sex unknown and assumed random). A pair refers to two samples collected from the same patellae of the same animal and cultured in the same well. All samples were cultured for a minimum of 15 hours (overnight) before testing for a consistent baseline, and all tests were done within 72 hours of sample collection. Preliminary tests showed cell viability and ORI metrics remained stable over 72 hours (Figs. S1–S2).

### Tensile Loading

**Fig. 1:**
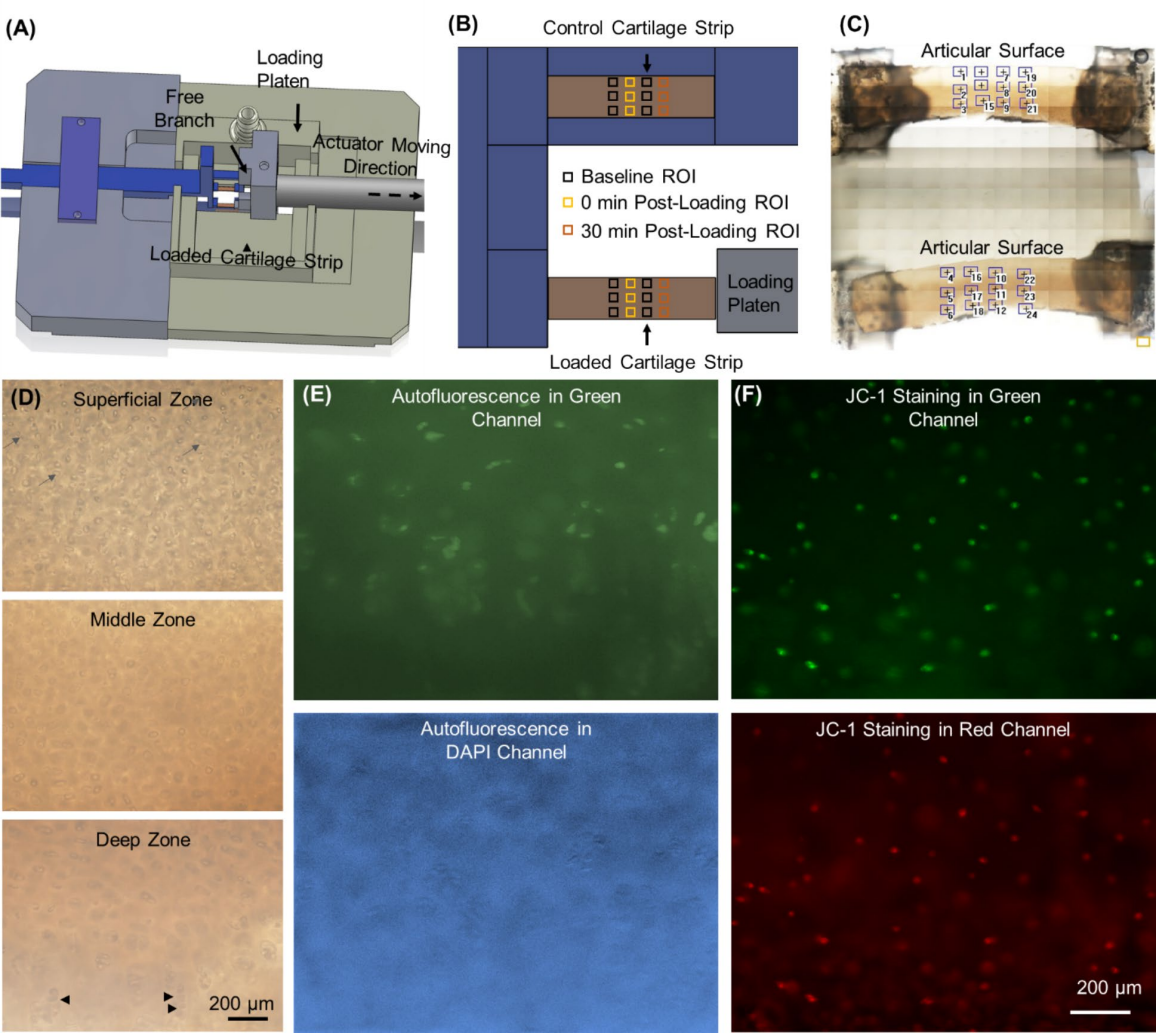
Schematic of loading device, regions of interest (ROIs), and representative images. **A** CAD representation of loading device; **B** schematic showing microscope view of ROIs; **C** tilescan of representative samples showing actual ROIs; **D** brightfield images showing three zones with elliptical cells in the superficial zone (arrows) and columns in the deep zone (arrow heads); **E** representative ORI autofluorescence images show cells visible in both channels (note that the DAPI channel had the background removal filter applied in ImageJ); **F** representative JC-1 images show cells visible in both channels.

Tensile loading was performed using a customized microscope-top loading device (Fig. 1A). Tensile loading was selected as the stimuli because it has been previously shown to induce changes in cartilage redox [9] and because it allows us to test samples with dimensions that enable ORI in all zones. One cartilage strip was placed between the backplate and the loading platen using a small amount of cyanoacrylate (Scotch Super Glue Gel) on each end. The loading platen was attached to an actuator (Aerotech ACT165D, Aerotech, Pittsburgh, Pennsylvania). The other strip was placed between the backplate and the free branch, serving as the control. Both strips were submerged in PBS at room temperature and oxygen concentration throughout the test. The strip connected to the loading platen was subjected to a single tensile loading at either 1.00 s^−1^ or 0.10 s^−1^ to a target engineering strain of 0.30. Nineteen pairs of strips were in the 1.00 s^−1^ group and 18 were in the 0.10 s^−1^ group. No preload was applied but samples were glued flat to minimize slack. Morphological integrity of loaded strips was checked at 4 × visually after each test.

The loading process of the 1.00 s^−1^ group was recorded by a high-speed camera (7000 frames per second, Phantom V1211 monochrome, Vision Research, Wayne, New Jersey) and that of the 0.10 s^−1^ group was recorded by the integrated microscope camera (20 frames per second, Olympus DP80, Center Valley, Pennsylvania). The achieved strain rate for each sample was calculated based on the displacement of the backplate and showed that the two groups were distinct from each other (Table S1).

### Optical Redox Imaging

Autofluorescent images were acquired at 20× before and after loading using an inverted epifluorescent microscope (Olympus IX-71), and imaging was not conducted during loading. Twelve regions of interest (ROI) were selected on each loaded sample and non-loaded counterpart (Fig. 1B), covering the superficial, middle, and deep zones. Cartilage zone was by eye based on distance from the articular surface and cell morphology. Before loading, images were taken in each baseline ROI in a random order (Fig. 1B). At 0 and 30 minutes after loading, one ROI was randomly selected from the corresponding 6 ROIs (Fig. 1B) and imaged. At each ROI, images were taken in the Green channel (excitation 470–490 nm, emission 500–550 nm, exposure time 833.33 ms), corresponding to the spectrum of FAD, and the DAPI channel (excitation 361–389 nm, emission 435–485 nm, exposure time 28.87 ms), corresponding to the spectrum of NAD(P)H.

### Mitochondrial Membrane Potential Measurement

Out of all loaded-control cartilage strip pairs, 16 pairs were subject to mitochondrial membrane potential dye staining (8 pairs from 0.10 s^−1^, 8 pairs from 1.00 s^−1^). After post-loading ORI was done, both strips were removed from the backplate for JC-1 dye (Invitrogen™) staining following manufacturer’s instructions. Briefly, samples were incubated with 10 µg/mL JC-1 dye for 15 minutes at 37 °C. Images were then taken in green and red fluorescent channels at each zone at 20×, corresponding to depolarized and polarized mitochondria, respectively. For samples that underwent testing including JC-1staining and imaging, the total test time was approximately 2 hours.

### Image Data Processing

ORI and mitochondrial membrane potential images were analyzed by measuring the average intensity of each image using the *mean()* function in Matlab (R2023b). Post-loading autofluorescence intensity in both green and DAPI channels was divided by the averaged pre-loading value of the same sample in corresponding zone. For JC-1 images, fluorescent intensity in the red channel was divided by that in the green channel in the same ROI. The R/G fluorescence ratio reflects MT polarization level.

### Statistical Methods

All statistics were done in Matlab (R2023b). A generalized linear mixed-effects model (GLMM) was run to determine the significant predictors to MT membrane potential. The dependent variable was JC-1 R/G ratio. The fixed effects including normalized green intensity, normalized DAPI channel intensity, loading rate, loading group (load vs. control, data from both groups was used), and zone. Only autofluorescence intensity measured in the second time slot (30 minutes after loading) was used because all JC-1 measurements were done shortly after that time point. All 2 way- and 3-way interactions were allowed. The random effect was sample, and an individual intercept was assigned for each sample.

The full dataset was then randomly split into training dataset (70% of the data) and testing dataset (30% of the data) using the *cvpartition()* function. Significant main effects and interactions found in the original GLMM were retained in a new model that was trained using a training dataset and evaluated using a testing dataset. After being trained using the training dataset, the model predicted MT R/G ratio for testing dataset. The Euclidean norm of the difference between predictions and actual values was calculated using the *norm()* function and divided by the number of samples in training data to get the averaged L2 norm.

The effects of mechanical stimuli on ORI metrics was also investigated using GLMM. The dependent variables were normalized green intensity and normalized DAPI channel intensity, respectively. The fixed effects were loading rate, loading group, zone, and post-loading time (T1 vs. T2, T1 was shortly after loading and T2 was 30 minutes post-loading), and all interactions were allowed. Sample was used as a random effect. In all models, categorical variables were effects-coded.

## Results

### ORI Metrics in Response to Mechanical Stimulus can Predict Corresponding Changes in MT Membrane Potential

Intensity in both channels, loading rate, and loading group were significant fixed effects in GLMM in prediction of JC-1 R/G ratio (Table 1). The positive estimated coefficients for main effects indicated that MT R/G increased with increasing intensity in both channels and with increasing loading rate. The loaded group had a higher intercept than the control group. All two-way interactions of these fixed effects were significant except for that between loading rate and loading group. Three-way interaction between green intensity, DAPI channel intensity, and loading rate, and random effect of sample were also significant. Although the superficial zone showed significance in some 2- and 3- way interactions, zone was not retained in the training model because it was not a significant main effect. The Akaike Information Criterion and Bayesian Information Criterion of the model are 91.31 and 219.53, respectively. The adjusted *R* squared value is 0.82.

**Table 1:** Significant effects and interactions for MT R/G ratio

Term	*P* value	Coefficient [confidence interval]
Intercept	0.04	− 10.65 [− 20.97, − 0.32]
Green	0.02	12.35 [1.67, 23.04]
DAPI	0.02	12.49 [1.83, 23.14]
Rate	0.02	16.81 [2.92, 30.70]
Group	0.03	9.918 [1.31, 18.53]
Green:DAPI	0.03	− 12.58 [− 23.71, − 1.46]
Green:Rate	0.02	− 18.01 [− 32.72, − 3.31]
DAPI:Rate	0.03	− 16.95 [− 31.93, − 1.97]
Green:Group	0.03	− 10.36 [− 19.59, − 1.13]
DAPI:Group	0.04	− 9.42 [− 18.58, − 0.27]
Green:DAPI:Rate	0.03	18.03 [2.14, 33.91]

**Fig. 2:**
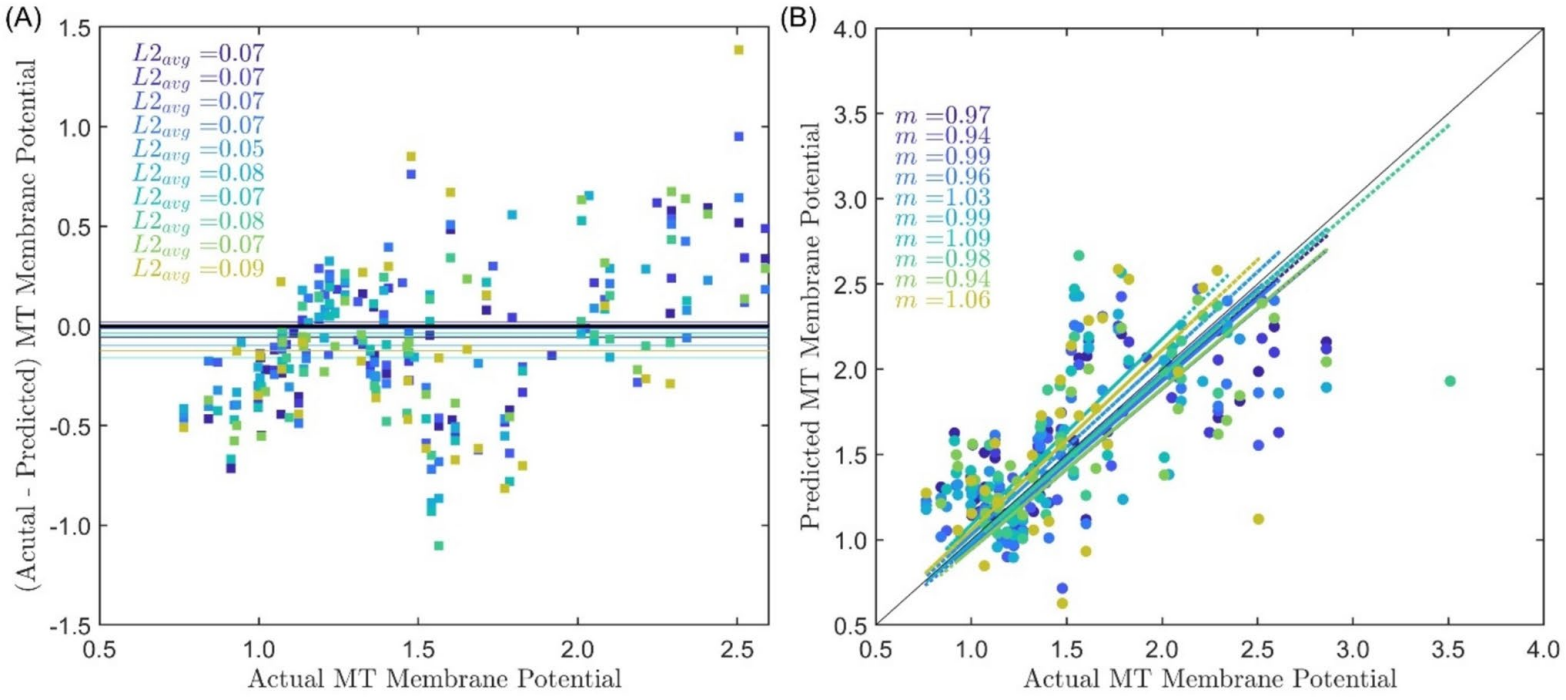
Prediction of MT depolarization from ORI metrics for 10 training datasets. **A** Actual MT R/G ratio vs. the difference between actual and predicted R/G ratio shows that MT R/G can be precited from ORI metrics. *L2*_*avg*_ is the averaged Euclidean norm (the black reference line shows a bias of zero, colored lines show the average bias for each corresponding dataset). **B** Actual MT R/G ratio vs. predicted R/G ratio; *m* is the slope for linear fitting (the black reference line shows a slope of 1.00, colored lines show the slope for each corresponding dataset).

GLMM that retained significant predictors resulted in an average difference between predicted and actual *R*/*G* ratio of 7.70 ± 1.06% across ten datasets (Table 2), in which the full dataset was randomly split for each. As visualized by the actual *R*/*G* ratio versus predictions and versus error plots, the model tended to underpredict MT R/G ratio, with an average slope of 0.95 ± 0.07 (Fig. 2).

**Table 2:** Errors in predicated MT membrane potential from ten testing datasets

Number of test	Averaged L2 norm
1	0.07
2	0.07
3	0.07
4	0.07
5	0.05
6	0.08
7	0.07
8	0.08
9	0.07
10	0.09
Average	0.07 ± 0.01

### Autofluorescence in Response to Mechanical Stimuli was Rate- and Zone- Dependent

**Fig. 3:**
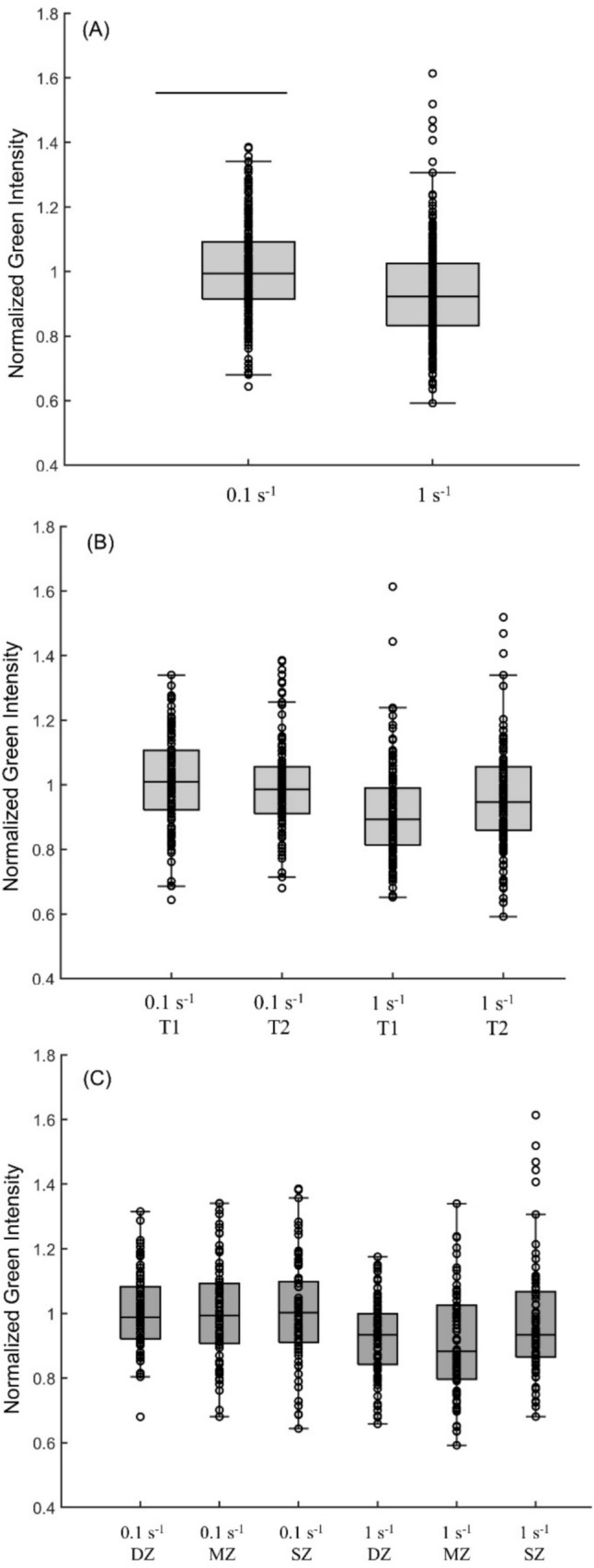
Visualization of significant effects on normalized green intensity. **A** Normalized green intensity of each sample grouped by strain rate (line indicates significant main effect of strain rate); **B** Normalized green intensity of each sample grouped by strain rate and time interaction; **C** Normalized green intensity of each sample grouped by strain rate and zone interaction.

Loading rate was the only significant main effect in the GLMM of normalized green intensity (Table 3). The negative estimated coefficient revealed that green intensity decreased with increasing loading rate (Fig. 3A). The interaction between loading rate and time was also significant. The negative value implied that the data in T1 had a steeper slope than that in T2 (Fig. 3B). The positive estimated coefficient of the interaction between loading rate and superficial zone was significant, implying that the slope of superficial zone data was flatter than the mean slope (Fig. 3C). No main effect was found significant in the GLMM of normalized DAPI channel intensity. Therefore, the 2- and 3-way interactions were omitted from interpretation.

**Table 3:** Significant effects and interactions for green intensity

Term	*P* value	Coefficient [confidence interval]
Intercept	0.00	1.01 [0.97, 1.06]
Rate	0.02	− 0.08 [− 0.14, − 0.01]
Rate: Zone_SZ	0.01	0.05 [0.01, 0.08]
Rate: time	0.03	− 0.03 [− 0.06, − 0.00]

## Discussion

This study demonstrated that MT membrane potential can be predicted by ORI metrics in cartilage explants. Tensile loading at two strain rates (1.00 s^−1^ and 0.10 s^−1^) was applied to induce changes in cartilage redox balance and strain rate was a significant effect in the GLMM. Prediction of MT R/G ratio suggests that ORI metrics and cellular redox balance are related in such a way that ORI metrics can be used to capture this important biological outcome. Given the body of literature that ties MT dysfunction to redox imbalance, these results indicate that ORI is predictive of redox balance. In addition to FAD and NAD(P)H intensity, optical redox ratio (FAD/(FAD + NAD(P)H)) could also be used to provide a global view of redox balance. However, in this study we analyzed FAD and NAD(P)H intensity separately to investigate the potential difference in the effect of loading to cartilage autofluorescence.

Loading rates used in this study (1.00 s^−1^ and 0.10 s^−1^) were selected to represent lower and higher bounds of physiological loading. Average cartilage contact deformation during 30%- and 100%-time during stance phase of gait was estimated to be 1.27 s⁻^1^ and 0.29 s⁻^1^, respectively. The estimation was done based on the deformation of medial compartment of tibiofemoral joint [22] and duration of stance phase in walking [23]. Normalized green intensity decreased with increased loading rate, which is consistent with prior studies showing that the mechanobiological response of cartilage is rate-dependent. For example, the percentage of chondrocytes exhibiting calcium signaling significantly increased after cyclic compression at 1.00 s^−1^, but not after cyclic compression at 0.25 s^−1^ or 0.50 s^−1^ [24]. Compared to low strain-rate compression, high strain-rate compression caused more cell death in the superficial zone [10]. In this study, the loading rate was not a significant effect in GLMM of normalized autofluorescence intensity in DAPI channel. Given that NAD(P)H is produced and consumed at multiple sites [25], it is likely that multiple pathways were affected by mechanical stimuli and the finding in this study was not conclusive in terms of the rate-dependency of NAD(P)H.

The difference in green intensity between the two loading rates was greater at T1 than T2, indicating a strong immediate response of redox balance to tensile loading that attenuated with time. This temporal profile of cartilage mechanobiological response was also documented in prior studies. Concentrations of multiple serum cartilage biomarkers significantly decreased 30 minutes after running or jumping exercise compared to that measured immediately after exercise [26]. The fraction of chondrocytes with depolarized mitochondria increased rapidly after traumatic injury and the rate of increase slowed down after about 15 min [27]. Similar to the measured rate effect, this study cannot conclude if NAD(P)H was insensitive to post-loading time.

Distinct mechanobiological response of cartilage in different zones was expected based on knowledge of zone-specific cell phenotypes. For example, a prior study by Ayala et al. revealed that cell death, cell apoptosis, and mitochondrial depolarization after impact loading and/or sliding was depth-dependent [28]. Zonal effects showed that the superficial zone displayed a flatter slope compared to the grand mean slope in the green intensity, which indicates lack of responsiveness in the superficial zone. This is in contrast to some prior studies that showed that the superficial zone was more mechanoresponsive than other zones [11, 28, 29]. Zonal difference in spontaneous calcium signaling is a potential way to explain the contradictory findings. In healthy human cartilage, a significantly larger portion of chondrocytes in the deep zone displayed stronger calcium signaling peaks compared to the superficial zone, while the middle zone had moderate peaks in the same recording time [30]. The zonal difference observed in this study was spontaneous when no loading was performed. Given that calcium signaling is essential for chondrocyte response to environmental stimuli including mechanical loading [31], and regulates loading-induced mitochondrial depolarization [32], it is possible that ORI metrics are regulated by calcium signaling and therefore showed a zonal difference that reflects those previously shown in calcium signaling.

Interestingly, loading group (load vs. control) was not a significant fixed effect in GLMM of autofluorescence intensity in either channel at either time point, despite the loading rate being a significant effect in the green channel. Given that both loaded and control samples were in the same solution throughout the test, the lack of statistical difference between loaded and control samples suggests that ORI metrics might be regulated by cartilage-to-cartilage crosstalk via diffusion. This would be consistent with a previous study reporting that mechanical loading-induced calcium signaling spread in chondrocytes that had no physical contact [33]. It is possible that similar mechanisms regulated ORI metrics and abolished the difference between loading and control groups. An alternate explanation is that mechanical stimuli induced adenosine triphosphate (ATP) release into the PBS, which could regulate cartilage mechanobiological response [34, 35]. Mechanical loading of chondrocyte-agarose constructs increased ATP concentration in PBS by 7 times in the loaded group compared to unloaded control [34]. Although the concentration decreased with time, it was still 2.5 times higher than control 30 minutes after loading. Similarly, a 20-minute loading at 0.33 Hz significantly increased medium ATP concentration when comparing to pre-loading condition [35].

It was worth noting that sample was significant as a random predictor in GLMM of MT membrane potential. The heterogeneous nature of chondrocyte metabolic response was also shown previously in spontaneous calcium signaling [36]. Despite the cell-to-cell variation, mechanical loading significantly increased the percentage of chondrocytes that displayed calcium signaling [24]. Hence, environmental stimuli such as mechanical loading have the potential to amplify the readings and should be integrated in the development of ORI as a clinical tool.

This study includes several limitations. All cartilage explants were collected from healthy juvenile pigs, so the relationship between ORI metrics and MT membrane potential must be further investigated in a wider age range and under various disease stages to fully understand its behavior. Another limitation was that cartilage strips were manually cut. The resulting inhomogeneity in strip thickness could introduce background fluorescence from out-of-focus cells. By normalizing the post-loading intensity to the pre-loading value of the same sample, sample-to-sample variation caused by different thickness was corrected. However, the thickness difference within each strip could still be confounding. ROIs were not repeatedly imaged because of photobleaching. Consistent cell density within each zone was assumed based on tissue source and health, however, the number of cells in each ROI was not quantified and this could introduce variation in measured intensity. In consideration of temporal changes in autofluorescence during tests, control and loading conditions were not applied to the same sample, which could contribute to variation. In addition, imaging was conducted in PBS, which may also affect cartilage. This choice was made to avoid any potential fluorescent signals from media.

A previous study from our lab [21] showed that autofluorescence intensity in both channels increased with age and degradation, both cases in which oxidative stress is known to present. It seems to conflict with the finding in this study, which shows a positive correlation between intensity and MT R/G ratio, as low R/G ratio is commonly related to mitochondrial dysfunction and oxidative stress. It is worth noting that there are a few differences between these two studies that could contribute to this discrepancy. Here we divided the post-loading intensity by the pre-loading value, while previous work reported positive correlations between raw intensity with age and degeneration. Plus, correlation observed in this study was mechanobiological response, while the previous study did not include mechanical stimuli and focused on basal difference in redox status.

In conclusion, this study showed that ORI metrics can predict the measurements of mitochondrial membrane potential made by an exogenous fluorescent probe. As a label-free, real-time imaging technique, ORI has the potential to be developed as a tool to evaluate cartilage redox balance in clinical settings. The responsiveness of ORI metrics, especially the green intensity, to strain rate, post-loading time, and cartilage zone highlighted its wide application. Despite the strong potential, implications of altered ORI in cartilage are still being explored. FAD and NAD(P)H participate in complex redox reaction networks and the precise connection between ORI and oxidative stress is yet to be established. To further use ORI as a tool to understand loading-induced temporal flux of redox balance in cartilage, a clearer interpretation of temporal profile of FAD/NAD(P)H is needed. Future studies should aim to understand the mechanisms underlying these responses, and to characterize ORI across species, age, and disease state.

## Supplementary Information

Below is the link to the electronic supplementary material.

Supplementary file1 (DOCX 99 kb)
