# Supplementary material for: Optical Redox Imaging Predicts Post-Loading Cartilage Mitochondrial Membrane Potential
Source: Ann Biomed Eng. 2025 Jun 27;53(9):2111–9. doi: 10.1007/s10439-025-03784-1 (PMC12390869; doi:10.1007/s10439-025-03784-1)
Supplement: Supplementary file 1 — Supplementary file1 (DOCX 99 kb) [file 10439_2025_3784_MOESM1_ESM.docx]

**Supplementary information for**

**Optical Redox Imaging Predicts Post-Loading Cartilage Mitochondrial Membrane Potential**

*Annals of Biomedical Engineering*

Jingyi Wang^1^, Greta E. Scheidt^2^, Corinne R. Henak,^1,2,3^

^1^Department of Mechanical Engineering, University of Wisconsin-Madison, Madison, WI

^2^Department of Biomedical Engineering, University of Wisconsin-Madison, Madison, WI

^3^Department of Orthopedics and Rehabilitation, University of Wisconsin-Madison, Madison, WI

*Address Correspondence to:

Corinne R. Henak, PhD

Associate Professor

3031 Mechanical Engineering Building

1513 University Ave

Madison, WI 53706

608-263-1619

[chenak@wisc.edu](mailto:chenak@wisc.edu)

**Confirmation of sample stability during culture**. To confirm that cultured cartilage remained viable and that ORI metrics were constant over time, hemicylinder cartilage explants were taken from two knees of a pig. Explants were cultured in the same condition as described in the method section of the manuscript. For viability, samples were incubated with 2 mL PBS with 1 μL calcein AM and 4 μL of EthD-III for 15 minutes at room temperature, then imaged in green (excitation 470 - 490 nm, emission 500 - 550 nm) and red (excitation 550 - 570 nm, emission 600 - 640 nm) fluorescent channel. Percent viable cells was calculated as the percentage of live cells (stained green) in total cells (stained red or green). Sample viability remained above 80% for 3 days and above 70% for four days (Figure S1). Despite showing slightly lower intensity on Day 0, ORI metrics remained constant for the following 3 days (Figure S2).

**Figure S1. Cell Viability from Day 0 to Day 4 after harvest.** The data corresponds to samples collected from left (DMEM L) and right (DMEM R) patella from one pig.

**Figure S2. ORI metrics from Day 0 to Day 4 after harvest.** The data corresponds to samples collected from the superficial, middle, and deep zone (SZ, MZ, and DZ, respectively) of the left and right patella from one pig.

**Outcomes of mechanical loading**. Achieved mechanical loading parameters were obtained from images acquired during loading. Data indicate that max force and strain were comparable between the two loading rates, but that the achieved strain rate was different (Table S1).

**Table S1. Achieved mechanical parameters of each loading rate group.**

| **Group** | **Max Strain (%)** | **Time at Max Strain (s)** | **Avg Strain Rate (s^-1^)** | **Max Backplate Displacement (mm)** | **Time at Max Displacement = Time at Max Force (s)** | **Max Force (N)** |
| --- | --- | --- | --- | --- | --- | --- |
| 1.0 s^-1^ | 17.91% ± 5.63% | 0.26 ± 0.07 | 0.71 ± 0.13 | 0.55 ± 0.28 | 0.25 ± 0.07 | 4.80 ± 2.41 |
| 0.1 s^-1^ | 19.64% ± 9.57% | 2.48 ± 0.86 | 0.07 ± 0.02 | 0.52 ± 0.22 | 1.90 ± 0.48 | 4.51 ± 1.91 |

**Positive control of mitochondrial depolarization.** To confirm that measuring average red/green ratio in the whole field is sufficient to reflect mitochondrial depolarization, FCCP was used to induce mitochondrial depolarization. Two cartilage strips were incubated in FCCP (10 µg/mL in culture media) for 40 minutes at 37 ℃, while two other strips were incubated in culture media for the same time period at the same temperature. JC-1 staining was performed on all strips as described in main text. Although the sample number was small and there was sample-to-sample variation in Red/Green fluorescence ratio, the FCCP group and control group was distinct from each other.


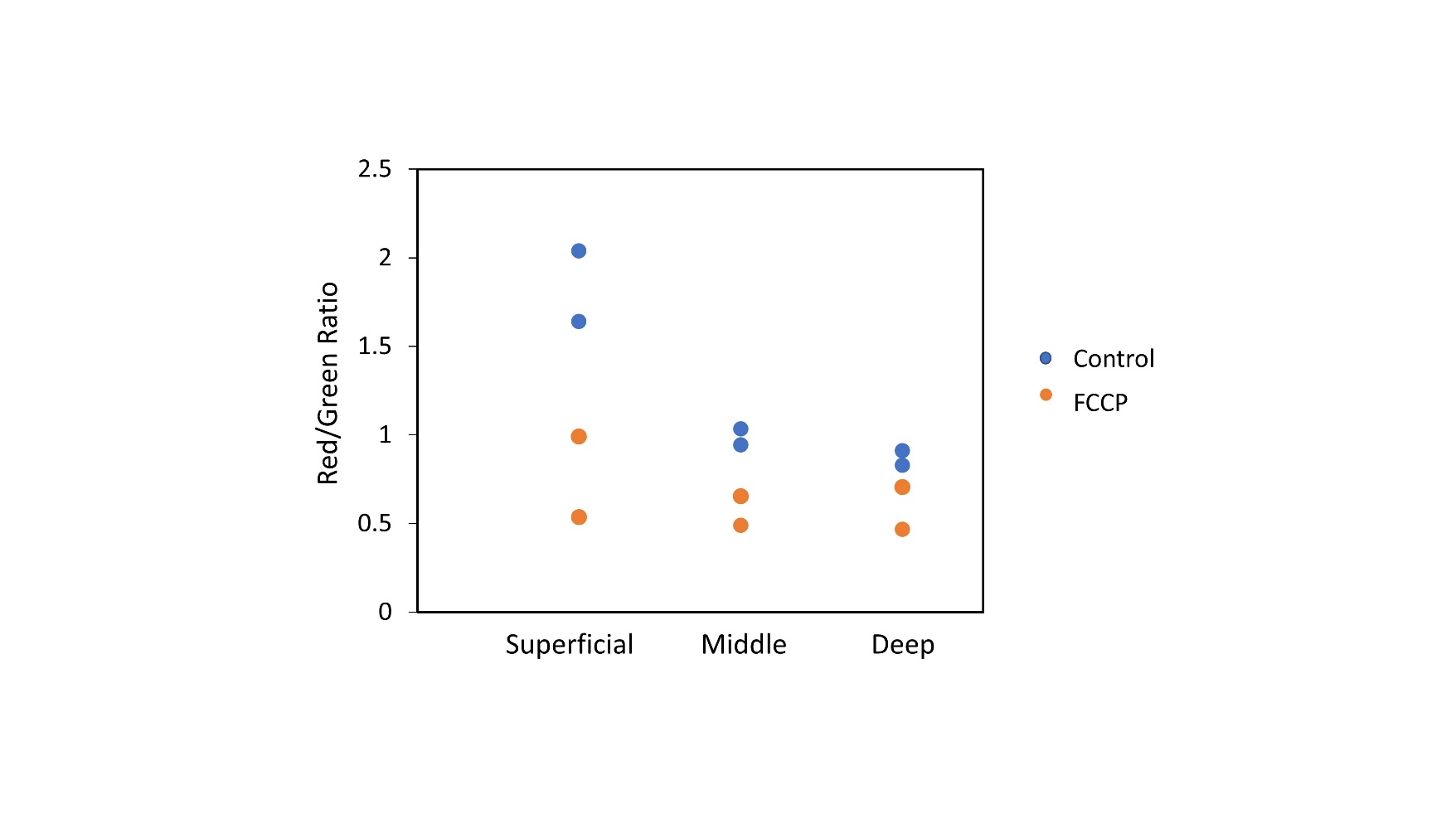


**Figure S3. Red/Green intensity ratio of FCCP incubated sample and control sample**.

**Time record for a subset samples**. Time recorded when each step was done. The time duration for each step was recorded for the first two samples to confirm that it was consistent (Table S2).

**Table S2. Time log for selected samples.**

| **Sample ID** | **Time Duration from out of Incubator till Baseline Imaging (hh:mm)** | **Time Duration from out of Incubator till Loading (hh:mm)** | **Total Time**  **(hh:mm)** |
| --- | --- | --- | --- |
| 11151 | 0:28 | 0:40 | 1:28 |
| 11152 | 0:31 | 0:49 | 1:24 |
| 11153 |  | 0:42 | 1:23 |
| 11161 |  | 0:42 | 1:26 |
| 11162 |  | 0:35 | 1:18 |
| 11163 |  | 0:40 | 1:26 |
| 03081 |  | 0:47 | 1:32 |

**Matlab code used for statistics.**

%% GLMM for MT R/G

data = readtable("MT-correlation-w cat var.xlsx",'Sheet','for_matlab');

LR100 = find(data.LoadingRate_s__1_==1);

LR10 = find(data.LoadingRate_s__1_==0.1);

SZ = find(data.Zone == "SZ");

MZ = find(data.Zone == "MZ");

DZ = find(data.Zone == "DZ");

loaded = find(data.LoadCtrlCode == 1);

ctrl = find(data.LoadCtrlCode == -1);

LR100SZ = find(data.Zone == "SZ" & data.LoadingRate_s__1_==1);

LR100MZ = find(data.Zone == "MZ" & data.LoadingRate_s__1_==1);

LR100DZ = find(data.Zone == "DZ" & data.LoadingRate_s__1_==1);

LR10SZ = find(data.Zone == "SZ" & data.LoadingRate_s__1_==0.1);

LR10MZ = find(data.Zone == "MZ" & data.LoadingRate_s__1_==0.1);

LR10DZ = find(data.Zone == "DZ" & data.LoadingRate_s__1_==0.1);

tbl = table(data.R_G,data.Green,data.DAPI,data.LoadingRate_s__1_,data.Zone,data.SampleN,data.LoadCtrlCode,...

'VariableNames',{'RG','Green','DAPI','Rate','Zone','Sample','Group'});

formula_RG = 'RG ~ Green * DAPI * Rate * Zone * Group + (1|Sample)';

lme_RG = fitglme(tbl,formula_RG,"DummyVarCoding","effects")

rSquared = lme_RG.Rsquared;

%% Re-run ten times to try fit with different train/test sets

ColorMtx = parula(13);

for i = 1:10 % adjust this index

C = ColorMtx(i,:);

%% GLMM with significant effects and interactions; Rerun this section to get another random split

n = size(data,1);

hpartition = cvpartition(n,'Holdout',0.30); % reserves 30% of data for testing

idxTrain = training(hpartition);

idxTest = test(hpartition);

tblTest = table(data.R_G(idxTest),data.Green(idxTest),data.DAPI(idxTest),data.LoadingRate_s__1_(idxTest),data.Zone(idxTest),data.SampleN(idxTest),data.LoadCtrlCode(idxTest),...

'VariableNames',{'RG','Green','DAPI','Rate','Zone','Sample','Group'});

tblTrain = table(data.R_G(idxTrain),data.Green(idxTrain),data.DAPI(idxTrain),data.LoadingRate_s__1_(idxTrain),data.Zone(idxTrain),data.SampleN(idxTrain),data.LoadCtrlCode(idxTrain),...

'VariableNames',{'RG','Green','DAPI','Rate','Zone','Sample','Group'});

formula_RG = 'RG ~ Green + DAPI + Rate + Group +Green*DAPI + Green*Rate + Green*Group + DAPI*Rate + DAPI*Group + Green*DAPI*Rate + (1|Sample)';

lme_RGTrain = fitglme(tblTrain,formula_RG)

yfit = predict(lme_RGTrain,tblTest);

figure(1); hold on

plot(data.R_G(idxTest),(data.R_G(idxTest)-yfit),'s','Color',C,'MarkerFaceColor',C)

xtickformat('%,.1f')

ytickformat('%,.1f')

xlabel('Actual MT Membrane Potential','Interpreter','latex')

ylabel('(Acutal - Predicted) MT Membrane Potential','Interpreter','latex')

hold on

hline = refline(0,mean(data.R_G(idxTest)-yfit)); hline.Color = (C);

hline = refline(0,0); hline.Color = ('k');

L2 = norm(data.R_G(idxTest)-yfit) % L2-norm

L2avg = L2/sum(idxTest)

axis([0.5 2.6 -1.5 1.5])

box on

text(0.75, (1.48 -0.12*i) ,['$L2_{avg} = $',num2str(L2avg,'%.2f')],'Interpreter','latex','Color',C)

figure(2); hold on

plot(data.R_G(idxTest),yfit,'o','Color',C,'MarkerFaceColor',C)

xtickformat('%,.1f')

ytickformat('%,.1f')

xlabel('Actual MT Membrane Potential','Interpreter','latex')

ylabel('Predicted MT Membrane Potential','Interpreter','latex')

dlm = fitlm(data.R_G(idxTest),yfit,'Intercept',false);

plot(data.R_G(idxTest),dlm.Fitted,':','Color',C)

box on

text(0.6, (3.6 - 0.12*i) ,['$m = $',num2str(dlm.Coefficients.Estimate,'%.2f')],'Interpreter','latex','Color',C)

axis([0.5 4.0 0.5 4.0])

hline = refline(1,0); hline.Color = ('k');

end
